# Emersion and Relative Humidity Modulate Stress Response and Recovery Dynamics in Juvenile Mussels (*Perna canaliculus*)

**DOI:** 10.3390/metabo11090580

**Published:** 2021-08-27

**Authors:** Natalí J. Delorme, David J. Burritt, Norman L. C. Ragg, Paul M. South

**Affiliations:** 1 Cawthron Institute, Private Bag 2, Nelson 7042, New Zealand; norman.ragg@cawthron.org.nz (N.L.C.R.); paul.south@cawthron.org.nz (P.M.S.); 2 Department of Botany, University of Otago, P.O. Box 56, Dunedin 9054, New Zealand; david.burritt@otago.ac.nz

**Keywords:** green-lipped mussel, Greenshell™ mussel, *Perna canaliculus*, spat, emersion, oxidative stress, reoxygenation stress, recovery, survival

## Abstract

The early stages of intertidal mussels, including the green-lipped mussel, *Perna canaliculus*, face both direct and indirect environmental threats. Stressors may influence physiological status and, ultimately, survival. An understanding of the nature of stress experienced is critical to inform conservation and aquaculture efforts. Here, we investigated oxidative stress dynamics in juvenile *P. canaliculus* in relation to emersion duration (1–20 h) and relative humidity (RH, 29–98%) by quantifying oxidative damage (protein carbonyls, lipid hydroperoxides, 8-hydroxydeoxyguanosine) and enzymatic antioxidants (superoxide dismutase, catalase, glutathione peroxidase and reductase). Mussels held in low RH during emersion experienced severe water loss (>70%), high mortality (>80%) and increased oxidative damage (35–45% increase compared to control conditions), while mussels held at high RH were not impacted, even after 20 h of air exposure. Following re-immersion, reoxygenation stress resulted in further increases in damage markers in mussels that had experienced dryer emersion conditions; protective action of antioxidants increased steadily during the 10 h re-immersion period, apparently supporting a reduction in damage markers after 1–5 h of immersion. Clearly, conditions during emersion, as well as duration, substantially influence physiological performance and recovery of juvenile mussels. Successful recruitment to intertidal beds or survival in commercial aquaculture operations may be mediated by the nature of emersion stress experienced by these vulnerable juveniles.

## 1. Introduction

Emersion is a significant source of stress for marine organisms. During emersion, organisms can be exposed to fluctuations in temperature, irradiance, and relative humidity (RH), with many intertidal organisms having physiological and behavioural adaptions that allow them to cope with such stressors [1,2]. For example, mussels can tolerate heat exposure and prevent desiccation by modifying their gaping behaviour, metabolism, and respiration [3,4,5,6,7]. The capacity to tolerate emersion in marine invertebrates closely relates to their bathymetric distribution [8,9]. Some bivalve molluscs depress their metabolism during emersion [10] or rely on anaerobic pathways to maintain ATP production for short emersion periods [11,12]; air-gaping during long-term emersion exposure may subsequently assist in acid-base regulation [13]. These different strategies to deal with emersion-related hypoxia are then likely to affect the organism’s responses following re-immersion in seawater.

Immersion in seawater after an emersion period is critical for the recovery and survival of the organism but is itself a stressor due to the oxidative damage caused to macromolecules (lipids, proteins and DNA) by rapid reoxygenation of the cells and the accumulation of free radicals and reactive oxygen species (ROS) in the cells [14,15]. ROS are produced in the cell through normal metabolism, and in molluscs they represent around 1–3% of the consumed oxygen [16]. ROS are neutralised by enzymatic and non-enzymatic antioxidants [17]; however, under stressful conditions, levels/activities of cellular antioxidants can be too low to cope with the production of ROS, resulting in oxidative damage and, ultimately, cell death [14,15,17]. Due to physical limitations to oxygen uptake during periods of air exposure, emersion often does not result in an immediate increased in oxidative damage [18]. However, the oxygenation of haemolymph in bivalves that had previously experienced emersion increased rapidly within the first hour of re-immersion in seawater [19]. This reoxygenation can result in a significant increase in ROS formation in bivalves, together with significant changes in oxidative damage and antioxidant levels/activities [20,21,22,23]. To date, most studies of the effects of emersion and recovery in marine bivalves have focused on adults [18,19,20,21,22,23]. Such effects possibly vary among life stages due to ontogenetic differences in metabolism, respiration, and behaviour, although few studies have reported emersion- and recovery-induced stress responses in juvenile marine invertebrates, e.g., [24,25].

Juvenile mytilid mussels (‘spat’) may be particularly vulnerable to the repercussions of emersion and reoxygenation stress. Settling juveniles typically colonise substrates free of adult mussels [26], which may reflect an area that is particularly affected by the stressors associated with emersion. Mussel spat also retain the capacity to resume pelagic drifting by the production of a mucus ‘parachute’, facilitating relocation but elevating the risk of predation [27]. It has been suggested that environmental stress may influence the resumption of pelagic drifting [28], and hence indirectly affect subsequent survival. The present study evaluated the stress response and recovery dynamics of juvenile, green-lipped mussels, *Perna canaliculus*, which is also an important aquaculture species in New Zealand [29,30]. The mussel industry routinely transfers juvenile mussels from their capture sites or from its single hatchery to marine farms around the country, a process that can involve emersion of up to 72 h [29,31]. Previous studies have described the stress response of *P. canaliculus* juveniles and adults to fasting, heat and simulated transport, showing that fasted juveniles are less able to tolerate subsequent stress, and that transport results in oxidative damage [32,33,34]. Additionally, variations in RH during emersion can affect the resettlement behaviour of juvenile *P. canaliculus* during recovery in seawater, although the underlying dynamic of physiological responses of these stressed juveniles remains unknown [28]. Therefore, the aim of this study was to assess the interactive effects of variations in emersion duration and RH on the stress responses and recovery dynamics of juvenile *P. canaliculus* during immersion in seawater. Stress responses were measured by quantifying oxidative damage (protein carbonyls, lipid hydroperoxides, 8-hydroxydeoxyguanosine) and enzymatic antioxidants (superoxide dismutase, catalase, glutathione peroxidase and reductase), as well as the water content and survival of the juvenile mussels. These parameters were evaluated experimentally in the laboratory to test the hypothesis that longer emersion times in a dryer environment would affect metabolism, stress levels, condition and recovery dynamics of juvenile *P. canaliculus*.

## 2. Results

### 2.1. Emersion Conditions

**Figure 1:**
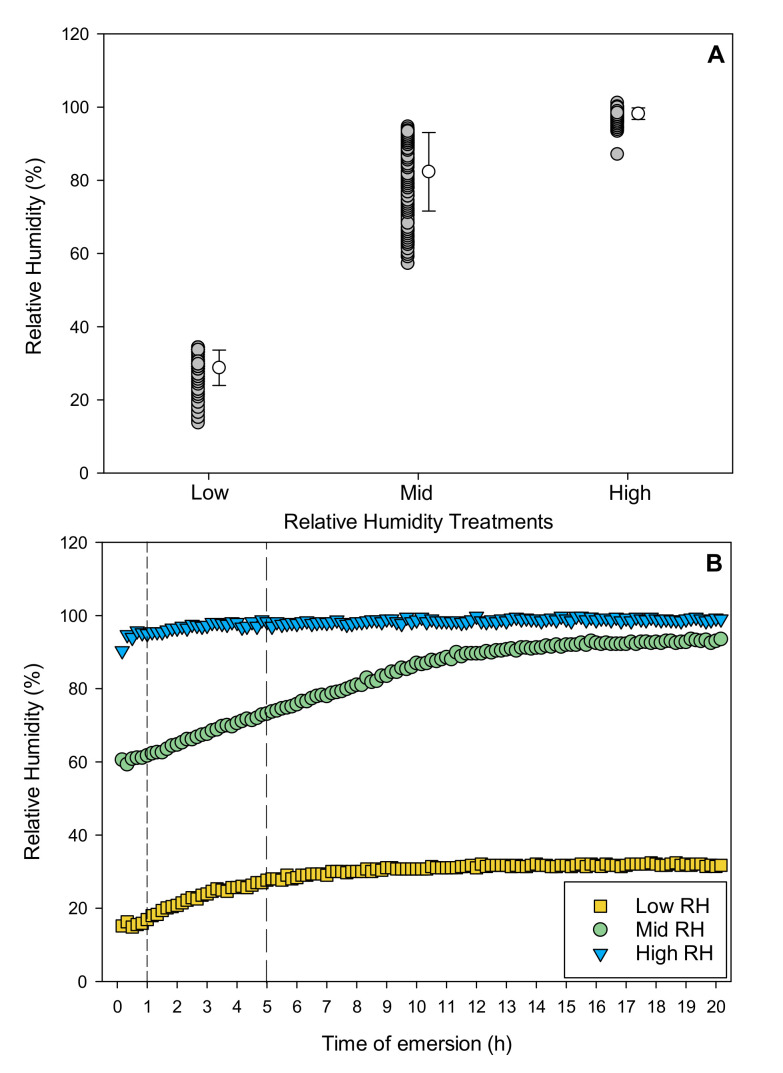
Relative humidity (RH, %; 18 °C) during emersion in treatments. (**A**): Plot showing data for each RH treatment (low, mid and high) and their respective means ± standard deviation (SD, *n* = 242). (**B**): Time series for each of RH treatment during 20 h of emersion (average of two loggers per treatment, 10 min sample interval). Short and long dashed lines show the end points of the 1 h and 5 h emersion treatments, respectively.

During emersion, relative humidity (RH) was consistently above 90% for the high RH treatment, with an average RH of 98 ± 2% (SD, *n* = 242; Figure 1A). In contrast, RH for low and mid RH treatments was more variable. Low and mid RH treatments started at approximately 15 and 60%, respectively, but average values over 20 h were, respectively, 29 ± 5% and 82 ± 11% (SD, *n* = 242 for each mean; Figure 1A). RH in the low RH treatment was 16 ± 2% during the first hour of emersion, increasing to 22 ± 4% after 5 h of emersion and then to a maximum value of around 32% by the end of the 20 h of emersion (Figure 1B). RH in the mid RH treatment was 61 ± 1% during the first hour of emersion and increased to 67 ± 4% after 5 h of emersion, before reaching a maximum value of around 94% by the end of the 20 h emersion period (Figure 1B).

### 2.2. Water Content

**Figure 2:**
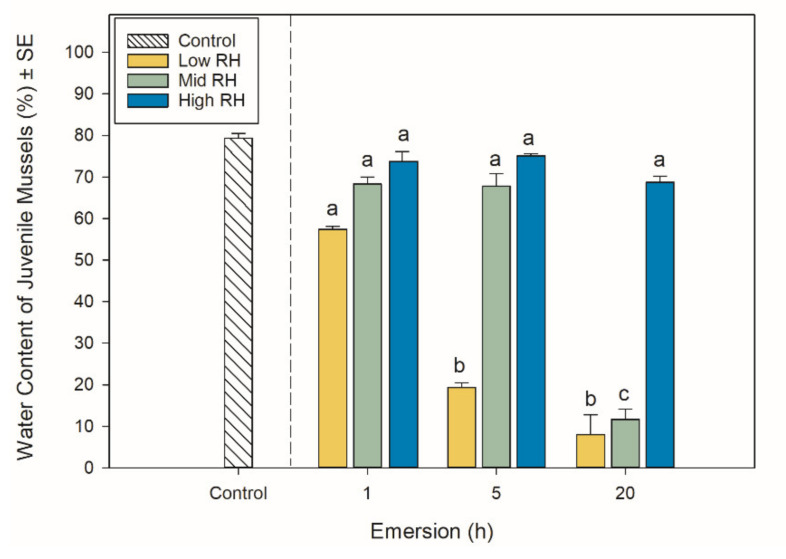
Water content (% live mass ± SE, *n* = 3) in juvenile *Perna canaliculus* exposed to different relative humidity (RH; low, mid, high) and emersion (1, 5, 20 h) treatments. Control bar indicates water content in juveniles that were not emersed (excluded from statistical analysis). Tukey pair-wise comparisons show significant differences (*p* < 0.05) for the interaction between emersion time and relative humidity treatments, which are denoted by different lower-case letters above bars.

The water content of the juvenile mussels was reduced by increasing emersion time and decreasing RH (Figure 2, Table 1). In the high RH treatment, water content was consistently high at around 68–75% across emersion treatments (Figure 2). The water content of mussels maintained at mid RH was high (~70%) and showed no change during the first 5 h of emersion, but then decreased to around 12% after 20 h of emersion (Figure 2). The water content of the juveniles in the low RH treatment decreased over time and was lower than the mid and high RH treatments after 1 and 5 h emersions (Figure 2). After 20 h of emersion, the water content of juveniles in the low and mid RH treatments was similar, with water content being between 8–12% (Figure 2).

### 2.3. Mortality Estimates: Observations and Staining

**Figure 3:**
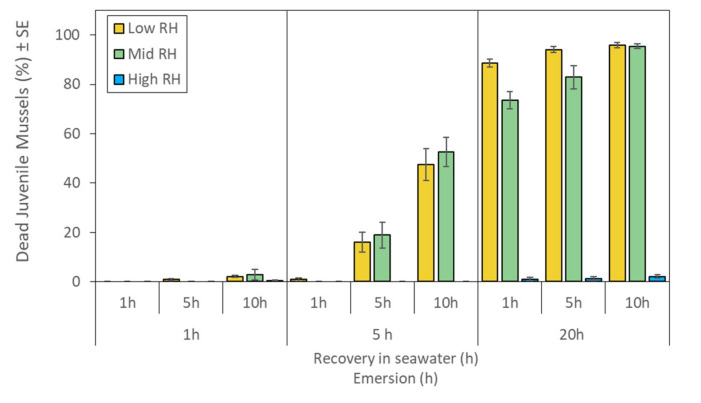
Estimates of mortality in juvenile *Perna canaliculus* exposed to different relative humidity (RH; low, mid, high) and emersion (1, 5, 20 h) treatments, followed by recovery in seawater (1, 5, 10 h). Data represent mean percent of dead juveniles ± standard error (SE, *n* = 5).

Observational live/dead assessments indicated interactive effects of emersion time, RH and recovery time on estimates of mortality that increased with time of emersion, especially in the low and mid RH treatments (Figure 3, Table 1). The effect of recovery time varied among emersion and RH treatments with the percentage of estimated dead mussels increasing over time in the low and mid RH treatments (Figure 3). Few juveniles that were emersed for 1 h or held at high RH appeared to die during this experiment (Figure 3). At the end of the experiment, 0.5 ± 0.32% (SE, *n* = 5) of juvenile mussels were estimated to be dead in control samples.

**Figure 4:**
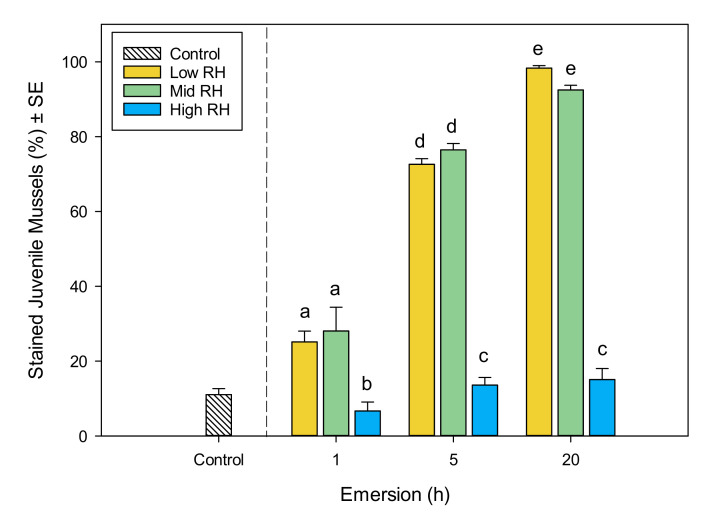
Fast Green staining of juvenile *Perna canaliculus* exposed to different relative humidity (RH; low, mid, high) and emersion (1, 5, 20 h) treatments, followed by 10 h recovery in seawater. Control bar shows percentage of stained mussels that were continuously immersed in flowing seawater (excluded from statistical analysis). Data represent the mean percent of stained mussels ± standard error (SE, *n* = 5). Tukey pair-wise comparisons show significant differences (*p* < 0.05) for the interaction between emersion time and relative humidity treatments, which are denoted by different lower-case letters above bars.

Fast Green staining in control mussels was apparent in 11 ± 1.6% (SE, *n* = 5) of individuals. The percentage of stained mussels increased with emersion time, with the greatest percentage occurring in mussels exposed to 20 h of emersion (Figure 4). A smaller percentage of juveniles stained in high RH treatments at all emersion durations relative to the mid and low RH treatments (Figure 4). Low and mid RH treatments had similar effects on the percentage of stained juveniles within each of the 1 and 5 h emersion treatments (Figure 4). After 20 h of emersion, there were fewer mussels stained in the mid RH treatment compared to the low RH treatment, but the percentage stained in these treatments was 84% greater than in the high RH treatment (Figure 4).

### 2.4. Oxidative Damage

There were interactive effects of emersion time, RH treatment and recovery time on levels of protein carbonyls (PCs), lipid hydroperoxides (LPs) and DNA damage, measured as 8-OHdG, in juvenile mussels (Table 2).

**Figure 5:**
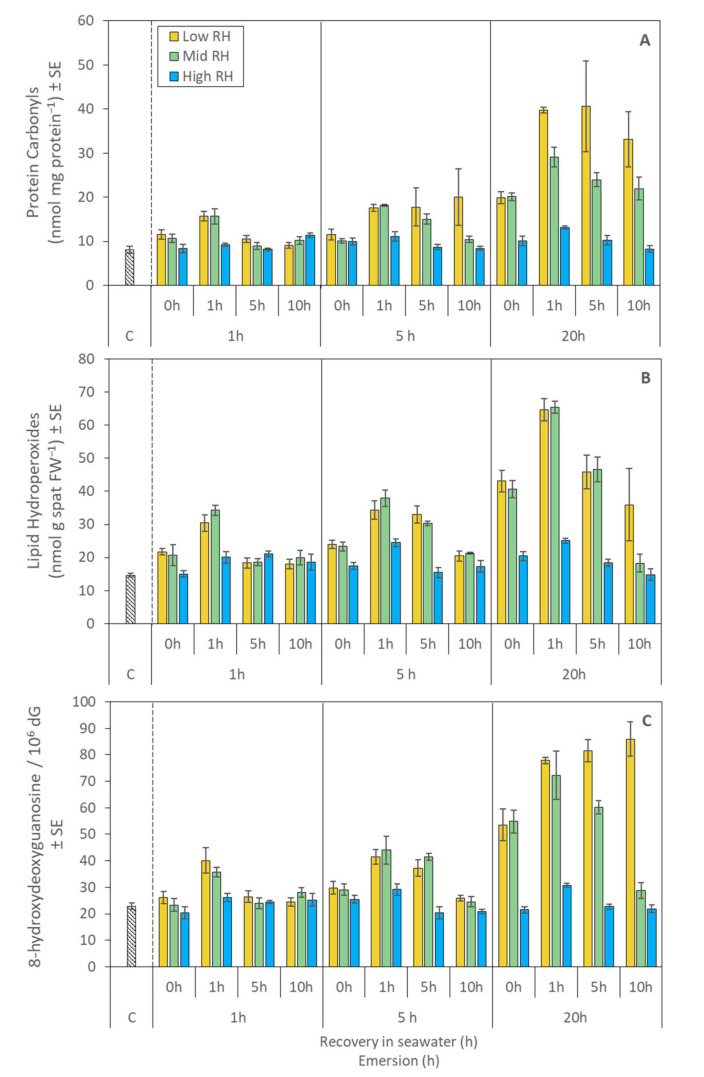
Oxidative damage biomarkers in juvenile *Perna canaliculus* exposed to different relative humidity (RH; low, mid, high) and emersion (1, 5, 20 h) treatments, followed by recovery in seawater (0, 1, 5, 10 h). Control bar (c) shows biomarker concentration in mussels that were continuously immersed in flowing seawater (excluded from statistical analysis). (**A**): Protein carbonyls (PCs); (**B**): Lipid hydroperoxides (LPs); (**C**): 8-hydroxydeoxyguanosine (8-OHdG). Data are mean concentration ± standard error (SE, *n* = 3).

There were strong effects of emersion and recovery duration on PCs levels in all but the high RH treatment (Figure 5A, Table 2). After 1 h of emersion, levels of PCs were higher for the low and mid RH treatments (relative to high RH) after 1 h of recovery, which declined to similar levels to those observed in high RH after 5 and 10 h of recovery (Figure 5A). After 5 h of emersion, levels of PCs in the low and mid RH treatments were significantly elevated after 0, 1 and 5 h of recovery, but then declined in the mid RH treatment to levels approaching baseline after 10 h of recovery (Figure 5A). An emersion time of 20 h caused a more substantial increase in PCs levels in the low and mid RH treatments; these were sustained over 10 h of recovery, while PCs levels in the high RH treatment remained at basal levels (Figure 5A). The low RH treatment generally induced greater and more variable PCs levels compared to mid and high RH (Figure 5A).

Levels of lipid hydroperoxides (LPs) were generally greater in mussels in the low and mid RH treatments, rapidly rising during the first hour of re-immersion to maximum levels that increased in correlation with emersion time (Figure 5B, Table 2). LP levels subsequently decreased with increasing time of recovery, approaching baseline after 10 h, except for mussels exposed to 20 h of emersion at low RH, where LP levels remained significantly elevated (Figure 5B).

There were similar patterns for 8-OHdG levels with a general trend of increased 8-OHdG levels in mussels after 1 h of recovery in seawater (Figure 5C, Table 2). An exception for this measure of DNA damage was that 8-OHdG continued to increase in the low RH/20-h emersion treatment during recovery, in part driving a strong interaction among the experimental factors (Figure 5C, Table 2).

### 2.5. Enzymatic Antioxidants

**Figure 6:**
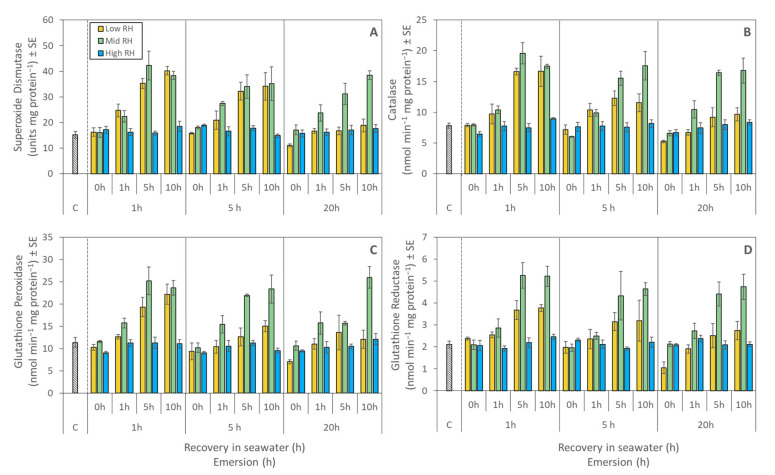
Antioxidant biomarker activity in juvenile *Perna canaliculus* exposed to different relative humidity (RH; low, mid, high) and emersion (1, 5, 20 h) treatments, followed by recovery in seawater (0, 1, 5, 10 h). Control bar (c) shows biomarker activity in mussels that were continuously immersed in flowing seawater (excluded from statistical analysis). (**A**): Superoxide Dismutase (SOD); (**B**): Catalase (CAT); (**C**): Glutathione Peroxidase (GPx); (**D**): Glutathione Reductase (GR). Data are mean concentrations ± standard error (SE, *n* = 3).

Enzymatic antioxidant activity was similar in all mussels sampled at the end of emersion, regardless of duration and RH treatment, resembling levels in control animals (Figure 6 A–D, Table 3). Activity consistently increased with recovery in seawater in low and mid RH treatments after 1 and 5 h of emersion, and in mid RH treatment after 20 h of emersion, driving RH × recovery interactions for all analyses (Figure 6 A–D, Table 3). Activity of enzymatic antioxidants for mussels from the high RH treatment remained at baseline levels during recovery, regardless of emersion duration (Figure 6 A–D).

## 3. Discussion

This study showed that the effects of emersion and the re-immersion dynamics of juvenile *Perna canaliculus* are complex and mainly depend on the conditions that the mussels experience during emersion. Juvenile mussels that experienced longer emersion at low and mid relative humidity (~15–60% RH) had increased water loss, increased oxidative damage and antioxidant enzymatic activity. These elevated levels, however, only tended to become apparent following re-immersion. The accumulation of oxidative damage in juvenile *P. canaliculus*, despite a corresponding increase in antioxidant activity, was correlated to increasing mortality rates during the 10 h re-immersion monitoring period (up to ~95% following 20 h emersion at low-mid RH). This correlation between emersion time, oxidative damage, and mortality was tested for all oxidative stress markers at the different RH levels at the end of the recovery time (see Supplementary Materials). The strongest correlation was observed for oxidative damage in the form of PCs at low and mid RH, where mortality increased steadily with PC levels, as time of emersion increased (*r*^2^ = 0.9991 at low RH and *r*^2^ = 0.7273 at mid RH). LPs and DNA damage also correlated to mortality observations only at low RH, but these correlations were weaker (*r*^2^ = 0.8630 and *r*^2^ = 0.7844 for LPs and DNA damage, respectively). This suggests that mussels that experience emersion at low RH are most likely to die due to oxidative stress, whereas oxidative damage is reduced at mid RH probably due to the specific action of antioxidants. At mid RH, it is likely that mussels were not dead but compromised, widely gaping and unable to close their valves after emersion and RH stress and are therefore classified as “dead”. Surviving mussels could also be physiologically impaired due to the high oxidative damage, potentially resulting in altered biological functions or even further mortality with increasing immersion time.

Green-lipped mussels naturally colonise rocks of the lower littoral and sub-tidal zones of New Zealand [35]. The emersion times used in this study are representative of a typical exposure period for an intertidal *P. canaliculus* (1 h), an exceptional low tide event (5 h), and an artificial emersion time which would represent a common transport time for mussel spat from hatchery to the grow-out farms (20 h). It should be noted that the juvenile mussels used in this study are hatchery produced from subtidal mussel populations (farmed); the results shown here might vary from the potential stress response of wild spat which may have previously experienced emersion [36] and associated stressors, with the potential to either increase [37] or reduce [32] subsequent stress tolerance.

Emersion of juvenile *P. canaliculus* caused severe water loss when RH was lower during emersion, and with increasing exposure times. Mussels isolate their soft tissues from the external environment by closing their valves during emersion, using periodic gaping behaviour to decrease their body temperature and facilitate gas exchange, allowing adult mussels to withstand long periods of emersion [4,6,38]. However, few studies have assessed the role of gaping in juvenile mussels. In the present study, there were two lines of evidence to suggest that juvenile *P. canaliculus* used gaping behaviour as a mechanism to reduce stress during emersion. First, there was a significant decrease in the water content of emersed juveniles that was exacerbated as emersion duration increased. Second, the mid humidity treatment showed increasing RH with the time of emersion, indicating that moisture from the mussels was released into the container during incubation. Even though RH increased in the mid RH treatment to levels ca. 80%, mussels experienced increased oxidative damage and antioxidant activity during the subsequent re-immersion period, compared to mussels held in high RH during emersion. This suggests that desiccation stress could also play a crucial role in the recovery dynamics of *P. canaliculus*.

It can be challenging to determine whether juvenile mussels are dead, moribund or alive using visual observations [28,39]. Here, our estimates suggested that mortality increased with recovery time, especially in the more severe RH treatments, with no differences between mid and low RH treatments at the end of the 10 h recovery period. Following 20 h emersion and low or mid RH, for example, >90% of individuals subsequently appeared unresponsive in water and took up Fast Green stain. However, measures of antioxidant enzyme activities suggest that these mussels were still metabolically active in the mid treatment. Indeed, given the substantial increases in antioxidant enzyme activities over the 10 h immersion period, it seems likely that many of these mussels were alive, but moribund and unable to respond to tactile stimulus or an osmotic shock (i.e., valve closure when immersed in freshwater and stain). This hypothesis could be tested with extended immersion periods to determine whether these mussels completely recover. By contrast, the high RH appears relatively benign, even when spat are emersed for 20 h, with most mussels showing signs of life or the ability to respond to tactile or osmotic stimulus and are therefore more likely to remain viable [39].

Marine littoral organisms experiencing natural emersion due to tidal cycles can accumulate modest levels of ROS during air exposure as they shift to anaerobic metabolism [17,40]. Despite demonstrating net metabolic depression [41], many enzymatic antioxidants are activated during the emersion period as a preparation for the reoxygenation stress (“preparation for oxidative stress”, POS) [42,43,44]. When organisms are immersed in the water for recovery, reoxygenation of haemolymph occurs rapidly (within one hour) [19], causing an oxidative burst, and the generation of large amounts of ROS [43]. In invertebrates, the oxidative burst is less intense and happens more slowly than in vertebrates; nonetheless, the excessive production of ROS can still result in oxidative damage [45]. In this study, juvenile *P. canaliculus* exposed to low and mid humidity air showed increased oxidative damage after 20 h of emersion compared to control mussels that remained submersed in seawater. Here, *P. canaliculus* experienced a more extreme emersion stress than most intertidal species as they moved from a completely subtidal environment to an extreme emersion period (20 h). However, oxidative stress was minimised if mussels were maintained in high humidity during emersion, where damage levels were similar to non-emersed control mussels. Reoxygenation has been shown to increase oxidative damage in the mussel *Mytilus edulis* [20], the gastropod *Crepipatella dilatata* [46], and the oyster *Crassostrea virginica* [47]. In the present study, reoxygenation stress resulted in a rapid increase in oxidative damage markers in all the humidity treatments, but levels were significantly higher following low and mid humidity exposure during emersion. Oxidative damage in the mussels that were exposed to high humidity during a 20 h emersion showed a decrease in damage to baseline levels after the mussels had been in seawater for 10 h.

Antioxidant enzyme activity in juvenile *P. canaliculus* did not increase during the emersion period at any humidity level. This suggests that juvenile *P. canaliculus* may have a reduced POS (i.e., preparation for oxidative stress) capacity to cope with reoxygenation stress during recovery, as seen in other invertebrate species [43]. In adults of the brown mussel, *Perna perna*, emersion stress for 48 h lowered the activity of enzymatic antioxidants; however, the levels of the non-enzymatic antioxidant glutathione (GSH) showed a rapid and persistent increase during emersion [48]. In the mussel *Mytilus edulis*, 48 and 72 h anoxia in seawater had little effect on the activity of antioxidants; however, antioxidant activity was suppressed after 72 h anoxia followed by 24 h of reoxygenation [20]. In the present study, there was an increase in enzymatic antioxidant activity of juvenile *P. canaliculus* in all treatments following re-immersion, which agrees with similar findings in other invertebrate species [46,49,50,51]. Levels of antioxidant activity in mussels held in high humidity conditions during emersion returned to baseline levels after 10 h of recovery. In contrast, mussels held at mid humidity during emersion showed no indication of declining after 10 h in seawater. It should be noted that mussels held at low humidity during emersion typically displayed low levels of antioxidant activity, similar to mussels that were held at high humidity. However, this result is likely to be an artifact of the high mortality of the mussels in the low humidity treatment, associated with high oxidative damage in the mussels sampled, but low antioxidant activity which is likely to have come from the small proportion of live mussels.

In this study, the increased action of antioxidants observed after short emersion periods (1 and 5 h) helped maintain relatively low levels of oxidative damage at all RH levels after re-immersion. However, at longer emersion (20 h), the activity of some antioxidants becomes compromised, resulting in an accumulation of oxidative damage in the tissues.

Emersion can be an occasional or regular event for juvenile *P. canaliculus*. For example, *P. canaliculus* can be an intertidal organism that experiences semidiurnal low tides, or they can be occasionally cast ashore while attached to drift algae. Juvenile *P. canaliculus* are also routinely emersed to transfer them to sea-based nursery farms for aquaculture, a process that can take as long as 3 days and be highly variable in terms of environmental conditions [52]. Losses of juvenile *P. canaliculus* after they are seeded onto a marine farm are a common problem for the mussel industry in New Zealand, where most of the juveniles are lost during the first few months of aquaculture [53,54,55,56]. Based on the results of the present study, it is possible that conditions in which the juvenile mussels are transported trigger a series of molecular, biochemical and physiological responses in the mussels that could have carry-over effects for the mussels after seeding, potentially affecting retention and survival of the juveniles. For example, juvenile resettlement behaviour was slowed and reduced by lower RH conditions during emersion [28]. The data presented here suggest that such impacts on behaviour could correspond to the physiological condition of the juveniles which, in the case of juveniles emersed in drier conditions, reflect increased ROS damage and antioxidant activity.

Complex, multi-species mussel guilds occupy the New Zealand rocky shore, with lower-littoral *Perna canaliculus* giving way to *Mytilus*, *Aulacomya* and *Xenostrobus* species on the higher shore [35,57]. Thermotolerance has been demonstrated to be a key determinant of this vertical zonation [57], which is turn likely to be influenced by region, latitude and genetic structure [58]. Based on the findings presented here, it would be valuable to consider the role of juvenile emersion and, in particular, reoxygenation stress as factors influencing natural distribution in an increasingly marine heatwave-prone region [59].

Overall, the present study showed that juvenile *P. canaliculus* are extremely sensitive to low humidity and prolonged emersion times, with mussels held in low RH during emersion experiencing severe water loss, high oxidative damage and high mortality, while mussels held at high RH were not impacted, even after 20 h of air exposure. These findings have significant implications for natural shoreline distribution and the aquaculture industry where juvenile mussels are routinely emersed during the production cycle. For aquaculturists, transportation methods should aim to maintain mussels in a high humidity environment at a constant temperature for the shortest possible time to mitigate the deleterious effects described in this study. When the time of emersion cannot be shortened, mussels should be held in a high relative humidity environment to minimise the stress responses elicited in the juvenile mussels, including increased oxidative damage and subsequent mortality. Further research is required to unravel the complex factors influencing resilience and environmental stress in the juveniles that appear to represent a major life-stage bottleneck for both cultured and wild *P. canaliculus* populations.

## 4. Materials and Methods

### 4.1. Experimental Design

Green-lipped mussel juveniles (~1 mm) were collected from a commercial hatchery (SPATnz) and transported to the laboratory of the adjacent Cawthron Aquaculture Park (Nelson, New Zealand). Mussels were weighed and separated into 178 circular sieves (8 cm diameter, 200 µm mesh size). Separate sets of experimental sieves were allocated for the determination of water content (27 sieves; ~120 mg of mussels in each sieve), assessments of survival (50 sieves; ~10 mg of mussels in each sieve) and oxidative biomarker analysis (111 sieves; ~1 g of mussels in each sieve). The sieves were then placed in a shallow acclimation tank with flowing seawater at 18 °C containing a mixture of axenically cultured microalgae (*Chaetoceros calcitrans*, *C. muelleri* and *Pavlova lutheri*). All sieves were supplied with food *ad libitum* during the first 24 h after collection before experimentation.

Experimental treatments consisted of three relative humidity (RH) treatments (low = ~15%, mid = ~60%, and high = ~90%), four emersion times (0, 1, 5 and 20 h) and three recovery times (1, 5 and 10 h) following re-immersion in seawater. Control mussels were not emersed during the experiment. Replication consisted of three replicated sieves per treatment for oxidative damage and antioxidant biomarker analyses, three replicated sieves for water content analysis, and five replicated sieves for survival. Lowered RH levels were achieved by adding different amounts of desiccant (silica gel) to a circular, 750 mL air-tight plastic container; for the high RH treatment, a seawater-saturated cotton cloth was added to the containers. Following acclimation, the experimental sieves with mussels were blot-dried and randomly allocated to the different RH treatments. All containers were tightly closed immediately after addition of the experimental sieves and placed in an incubator at 18 °C. RH loggers (Hygrochrons, iButtonLink Technology, Whitewater, WI, USA) sampling at 10 min intervals were added to 6 of the containers allocated to 20 h of emersion (two per RH treatment). After each of the emersion treatments, the mussels in the sieves were either sampled, assessed as described below, or randomly assigned to re-immersion treatments.

### 4.2. Water Content and Mortality Estimates

Water content in juvenile mussels was determined as percent mass loss by weighing (wet), drying (at 100 °C for 24 h), and re-weighing.

Sieves allocated to survival monitoring were surveyed repeatedly throughout the re-immersion recovery period. A stereomicroscope was used to observe the immersed mussels; wide-open individuals that did not respond to a tactile stimulus (prodding with forceps) were considered to be dead [25].

After the last recovery time point (10 h), mussels were stained using the Fast Green method [39], with minor modifications, to provide an indication of viability. In brief, mussels were transferred from the experimental sieves into freshwater (~20 mL) in 35 mL plastic containers to induce valve closure. One drop of concentrated Fast Green dye was added to each container, and the mussels were left in the stain for 1 h. Individuals that were incapable of sustained valve closure throughout this period were considered inviable (dead or moribund) and took up dye; the green-stained tissues of these spat could subsequently be discerned through the translucent valves. The mussels were subsequently rinsed and frozen at −20 °C. Samples were thawed and mussels were counted; the percentage of stained individuals per sample was calculated and used as the response variable in analyses.

### 4.3. Oxidative Damage

Juvenile mussels were sampled for oxidative biomarker analyses before emersion (control), directly after the completion of the emersion periods (0 h recovery) or after 1, 5 or 10 h of recovery in seawater. Three sub-samples of ~130–140 mg of juvenile mussels (fresh weight) were taken from each of the replicate sieves for each assay. Each sub-sample was placed into 2 mL cryo-vials, flash frozen in liquid nitrogen and stored at −80 °C until analyses. The sub-samples were used to determine oxidative damage (protein carbonyls (PCs), lipid hydroperoxides (LPs) and 8-hydroxydeoxyguanosine (8-OHdG)) and antioxidant enzyme activity (superoxide dismutase (SOD), catalase (CAT), glutathione peroxidase (GPx) and glutathione reductase (GR)).

#### 4.3.1. Macromolecule Extraction

Macromolecule extractions (protein, lipid and DNA) for determination of oxidative damage in juvenile *P. canaliculus* were performed according to Delorme et al. [32] In brief, total protein was extracted on ice by adding 900 µL of ice-cold enzyme extraction buffer (100 mM potassium phosphate [pH 7.5] containing 50 mM NaCl, 0.1 mM Na2EDTA, 1% polyvinylpyrrolidone−40, 2 mM phenylmethylsulfonyl fluoride and 0.1% TritonX−100) and homogenising for 30 s at 1500 rpm (1600 MiniG^®^, SPEX^®^) using zirconia/silica beads and a pre-chilled cryo-block (SPEX^®^). The samples were then centrifuged for 15 min at 17,000× *g* at 4 °C and the supernatant (i.e., protein extract) purified using ultrafiltration and purification columns (AMICON). The purified protein extract was then washed and reconstituted with 250 µL of 50 mM potassium phosphate buffer (pH 7.2), placed in a 1.5 mL microcentrifuge tube, blown with oxygen-free nitrogen and stored at −80 °C. Protein content was determined by the Lowry protein assay [60]. Samples were diluted with potassium phosphate as required before analysis. The levels of protein carbonyls (PCs) were determined via reaction with 2.4-dinitrophenylhydrazine (DNPH) as described by Reznick and Packer [61] and expressed as nmols of carbonyls mg of protein^−1^.

Total lipids were extracted by adding 600 µL of methanol:chloroform (2:1 *v*/*v*) and homogenised as described above. The homogenised sample was left to stand for 5 min and an extra 400 µL of chloroform were added and vortexed vigorously for 30 s. Then, 400 µL of MilliQ water were added and the sample vortexed again for 30 s. The samples were then centrifuged at an ambient temperature for 30 s at 17,000× *g*. Finally, the chloroform phase (bottom layer) was removed and transferred to a clean 1.5 mL microcentrifuge tube, blown with oxygen-free nitrogen and stored at −80 °C until analysis. The level of lipid hydroperoxides (LPs) in the samples was determined by absorbance at 500 nm using the ferric thiocyanate method described by Mihaljevic et al. [62], adapted for measurement in a microtitre plate reader. A calibration curve with t-butyl hydroperoxide was used and the LP content calculated as nmol of lipid hydroperoxide per mg of fresh (wet) mussel weight.

PC and LP assays were carried out using a Victor 1420 Multilabel plate reader (Perkin Elmer Wallac, Waltham, MA, USA) fitted with a temperature control cell (set to 25 °C) and an auto-dispenser. Data were acquired and processed using the WorkOut 2.0 software package (Perkin Elmer, Waltham, MA, USA).

DNA extraction was performed using an ISOLATE II Genomic DNA Kit (Bioline, Memphis, TN, USA), with one minor modification—the samples were crushed and homogenised using a tube pestle after the addition of the pre-lysis buffer. The final DNA extracts were placed in a 1.5 mL microcentrifuge tube, blown with oxygen-free nitrogen and stored at −80 °C until analyses. The level of oxidised DNA was calculated by quantifying the amount of 8-hydroxydeoxyguanosine (8-OHdG) present using high-performance liquid chromatography (HPLC) followed by UV detection of guanine and electrochemical detection (coulometric) of 8-OHdG as described previously for *P. canaliculus* juveniles [32].

#### 4.3.2. Enzymatic Antioxidants

The remaining protein extract was used to perform antioxidant enzyme assays: superoxide dismutase activity (SOD), catalase (CAT), glutathione peroxidase (GPx) and glutathione reductase (GR) as described by Delorme et al. [32] In brief, SOD was determined using a Cayman Chemicals Superoxide Dismutase Assay Kit and the activity expressed as units of SOD mg of protein^−1^. CAT was assayed using the chemiluminescent method of Maral et al. [63], as adapted by Janssens et al. [64] for 96-well microplates, and the activity expressed as µmol min^−1^ mg protein^−1^. GPx activity was measured according to the spectrophotometric method described by Paglia and Valentine [65] and expressed as nmol min^−1^ mg of protein^−1^. GR was assayed using the method of Cribb et al. [66], with minor modifications and activity expressed as nmol min^−1^ mg of protein^−1^. All enzymatic assays were carried out using a Perkin Elmer Wallac Victor 1420 multilabel counter (Perkin Elmer, Waltham, MA, USA) as detailed above.

### 4.4. Statistical Analyses

All analyses were carried out using analysis of variance (ANOVA) with α = 0.05 unless otherwise stated. Assumptions of ANOVA were checked using appropriate tests (Shapiro–Wilk, Brown Forsythe, Mauchly) and graphical observations. Water content, mortality and staining percentage data were arcsine-square root transformed prior to analysis. Water content data were not normally distributed but met the assumption of homoscedasticity and were analysed using a two-way ANOVA with RH level and emersion time as factors since balanced ANOVA is robust to deviations from normality [67]. Estimates of mortality data were analysed using a repeated measures ANOVA with RH and emersion time as the between-subjects effects, and re-immersion time as the within-subjects effect to account for repeated observations of individual sieves. Estimated mortality data were non-normal and variances were heterogeneous but met the assumption of sphericity and were analysed with α = 0.01 [68]. Staining data were analysed using a two-way ANOVA (α = 0.01), with RH level and emersion time as fixed factors. All oxidative damage (PCs, LPs, 8-OHdG) and enzymatic antioxidants (SOD, CAT, GPx, GR) data were analysed with three-way ANOVA using RH, emersion time, and recovery time as fixed factors. PC data did not meet parametric assumptions and were transformed to the reciprocal before analysis. Differences among treatments were identified using Tukey pair-wise tests with α = 0.05. Analyses were carried out using Sigma Plot 14.0 (SYSTAT Software, Inc., Chicago, IL, USA) or Statistica 12 (Statsoft) software.

## Figures and Tables

**Table 1:** ANOVA results of water content, mortality and staining percentage data for *Perna canaliculus* juveniles in different relative humidity (RH) treatments during emersion (E: 1, 5 and 20 h), followed by recovery in seawater (R: 1, 5 and 10 h). Degrees of freedom (df), mean square (MS), F-ratio and *p*-values are shown for each variable. Significant results (*p* < 0.05) are shown in bold.

**Water Content**	**df**	**MS**	**F**	***p***
Relative Humidity (RH)	2	0.565	32.785	**<0.001**
Emersion time (E)	2	0.276	16.023	**<0.001**
RH × E	4	0.077	4.515	**0.011**
Residual	18	0.017		
**Estimated Mortality**	**df**	**MS**	**F**	***p***
Relative Humidity (RH)	2	4.436	422.941	**<0.001**
Emersion time (E)	2	7.987	761.58	**<0.001**
RH × E	6	1.663	158.529	**<0.001**
Residual (between-effects)	36	0.01		
Recovery time (R)	2	0.776	159.186	**<0.001**
RH × R	4	1.662	30.832	**<0.001**
R × E	4	0.15	35.811	**<0.001**
RH × R × E	8	0.061	12.422	**<0.001**
Residual (within-effects)	72	0.005		
**Staining**	**df**	**MS**	**F**	***p***
Relative Humidity (RH)	2	2.142	246.423	**<0.001**
Emersion time (E)	2	1.454	167.251	**<0.001**
RH × E	4	0.209	24.026	**<0.001**
Residual	36	0.009		

**Table 2:** ANOVA results of oxidative damage biomarkers data for *Perna canaliculus* juveniles exposed to different relative humidity (RH) treatments during emersion (E: 1, 5 and 20 h), followed by recovery in seawater (R: 0, 1, 5 and 10 h). Significant results (*p* < 0.05) are shown in bold.

**Protein Carbonyls (PCs)**	**df**	**MS**	**F**	***p***
Relative Humidity (RH)	2	1.8^−2^	86.5	**<0.001**
Emersion time (E)	2	1.4^−2^	68.2	**<0.001**
Recovery time (R)	3	3.4^−3^	16.6	**<0.001**
RH × E	4	1.9^−3^	9.2	**<0.001**
RH × R	6	1.8^−4^	0.8	0.541
E × R	6	3.5^−4^	1.7	0.137
RH × E × R	12	7.3^−4^	3.5	**<0.001**
Residual	72	2.1^−4^		
**Lipid Hydroperoxides (LPs)**	**df**	**MS**	**F**	***p***
Relative Humidity (RH)	2	2026.9	87.2	**<0.001**
Emersion time (E)	2	2281	98.1	**<0.001**
Recovery time (R)	3	1371.2	59	**<0.001**
RH × E	4	521.8	22.4	**<0.001**
RH × R	6	151.4	6.5	**<0.001**
E × R	6	175	7.5	**<0.001**
RH × E × R	12	62.4	2.7	**0.005**
Residual	72	23.3		
**DNA Damage (8-OHdG)**	**df**	**MS**	**F**	***p***
Relative Humidity (RH)	2	4206.1	125.8	**<0.001**
Emersion time (E)	2	6239.2	186.6	**<0.001**
Recovery time (R)	3	1055.1	31.6	**<0.001**
RH × E	4	2001.9	59.9	**<0.001**
RH × R	6	277.5	8.3	**<0.001**
E × R	6	80.1	2.4	**0.036**
RH × E × R	12	198.6	5.9	**<0.001**
Residual	72	33.5		

**Table 3:** ANOVA results of enzymatic antioxidant biomarkers data for *Perna canaliculus* juveniles exposed to different relative humidity (RH) treatments during emersion (E: 1, 5 and 20 h), followed by recovery in seawater (R: 0, 1, 5 and 10 h). Significant results (*p* < 0.05) are shown in bold.

**Superoxide Dismutase (SOD)**	**df**	**MS**	**F**	***p***
Relative Humidity (RH)	2	1260.5	62.2	**<0.001**
Emersion time (E)	2	262.4	12.9	**<0.001**
Recovery time (R)	3	873.7	43.1	**<0.001**
RH × E	4	161.5	8	**<0.001**
RH × R	6	232.8	11.5	**<0.001**
E × R	6	36.2	1.8	0.115
RH × E × R	12	31.7	1.6	0.123
Residual	72	20.3		
**Catalase (CAT)**	**df**	**MS**	**F**	***p***
Relative Humidity (RH)	2	243.5	72.7	**<0.001**
Emersion time (E)	2	41	12.2	**<0.001**
Recovery time (R)	3	223.8	66.8	**<0.001**
RH × E	4	21.6	6.4	**<0.001**
RH × R	6	49.6	14.8	**<0.001**
E × R	6	4.5	1.4	0.248
RH × E × R	12	3.2	1	0.487
Residual	72	3.4		
**Glutathione Peroxidase (GPx)**	**df**	**MS**	**F**	***p***
Relative Humidity (RH)	2	521	66.4	**<0.001**
Emersion time (E)	2	61.6	7.9	**<0.001**
Recovery time (R)	3	308.2	39.3	**<0.001**
RH × E	4	21.9	2.8	**0.033**
RH × R	6	62.2	8	**<0.001**
E × R	6	10.7	1.4	0.239
RH F E × R	12	13.1	1.7	0.092
Residual	72	7.8		
**Glutathione Reductase (GR)**	**df**	**MS**	**F**	***p***
Relative Humidity (RH)	2	18.9	42.5	**<0.001**
Emersion time (E)	2	2	4.5	**0.015**
Recovery time (R)	3	13.2	29.8	**<0.001**
RH × E	4	1	2.3	0.067
RH × R	6	4.4	9.8	**<0.001**
E × R	6	0.2	0.5	0.811
RH × E × R	12	0.1	0.1	1
Residual	72	0.5		

## Data Availability

The data presented in this study are available on request from the corresponding author. The data are not publicly available due to the value of researchers’ interaction when/if the data are requested.

## Supplementary Materials

The following are available online at https://www.mdpi.com/article/10.3390/metabo11090580/s1, Figure S1: Correlations between oxidative damage and mortality observations after 10 h of recovery in seawater. A: Protein carbonyls (PCs); B: Lipid hydroperoxides (LPs); C: 8-hydroxydeoxyguanosine (8-OHdG; DNA damage). Data from different emersion times were combined to plot oxidative damage versus mortality across emersion time for each relative humidity (RH) treatment, Figure S2: Correlations between oxidative damage and mussel staining after 10 h of recovery in seawater. A: Protein carbonyls (PCs); B: Lipid hydroperoxides (LPs); C: 8-hydroxydeoxyguanosine (8-OHdG; DNA damage). Data from different emersion times were combined to plot oxidative damage versus staining across emersion time for each relative humidity (RH) treatment.
