# Supplementary material for: Emersion and Relative Humidity Modulate Stress Response and Recovery Dynamics in Juvenile Mussels (Perna canaliculus)
Source: Metabolites. 2021 Aug 27;11(9):580. doi: 10.3390/metabo11090580 (PMC8468153; doi:10.3390/metabo11090580)

metabolites

MDPI

Supplementary Material

Figure S1. Correlations between oxidative damage and mortality observations after 10 h of recovery in seawater. (**A**): Protein carbonyls (PCs); (**B**): Lipid hydroperoxides (LPs); (**C**): 8-hydroxydeoxyguanosine (8-OHdG; DNA damage). Data from different emersion times were combined to plot oxidative damage versus mortality across emersion time for each relative humidity (RH) treatment.

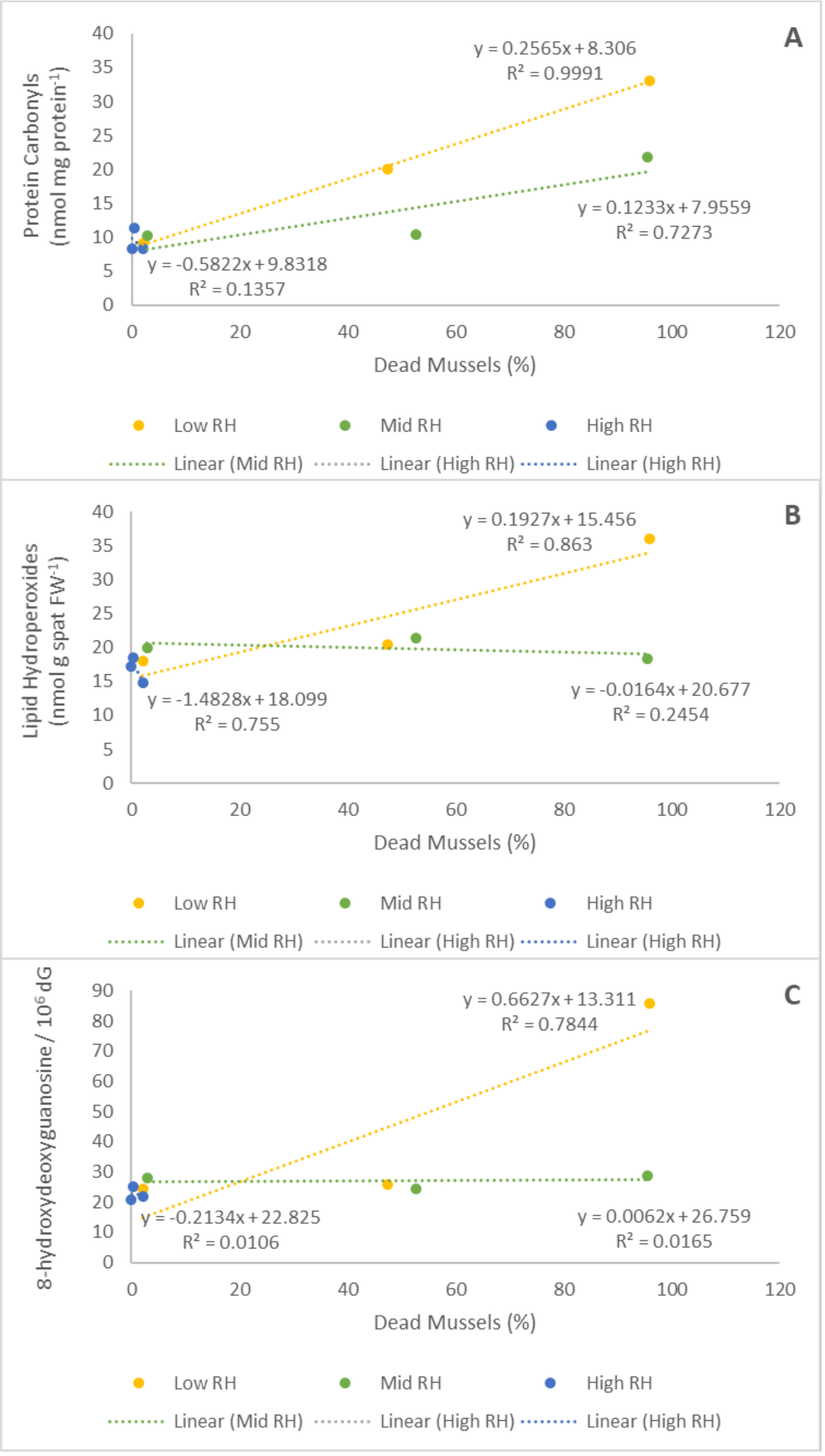

Figure S2. Correlations between oxidative damage and mussel staining after 10 h of recovery in seawater. (**A**): Protein carbonyls (PCs); (**B**): Lipid hydroperoxides (LPs); (**C**): 8-hydroxydeoxyguanosine (8-OHdG; DNA damage). Data from different emersion times were combined to plot oxidative damage versus staining across emersion time for each relative humidity (RH) treatment.

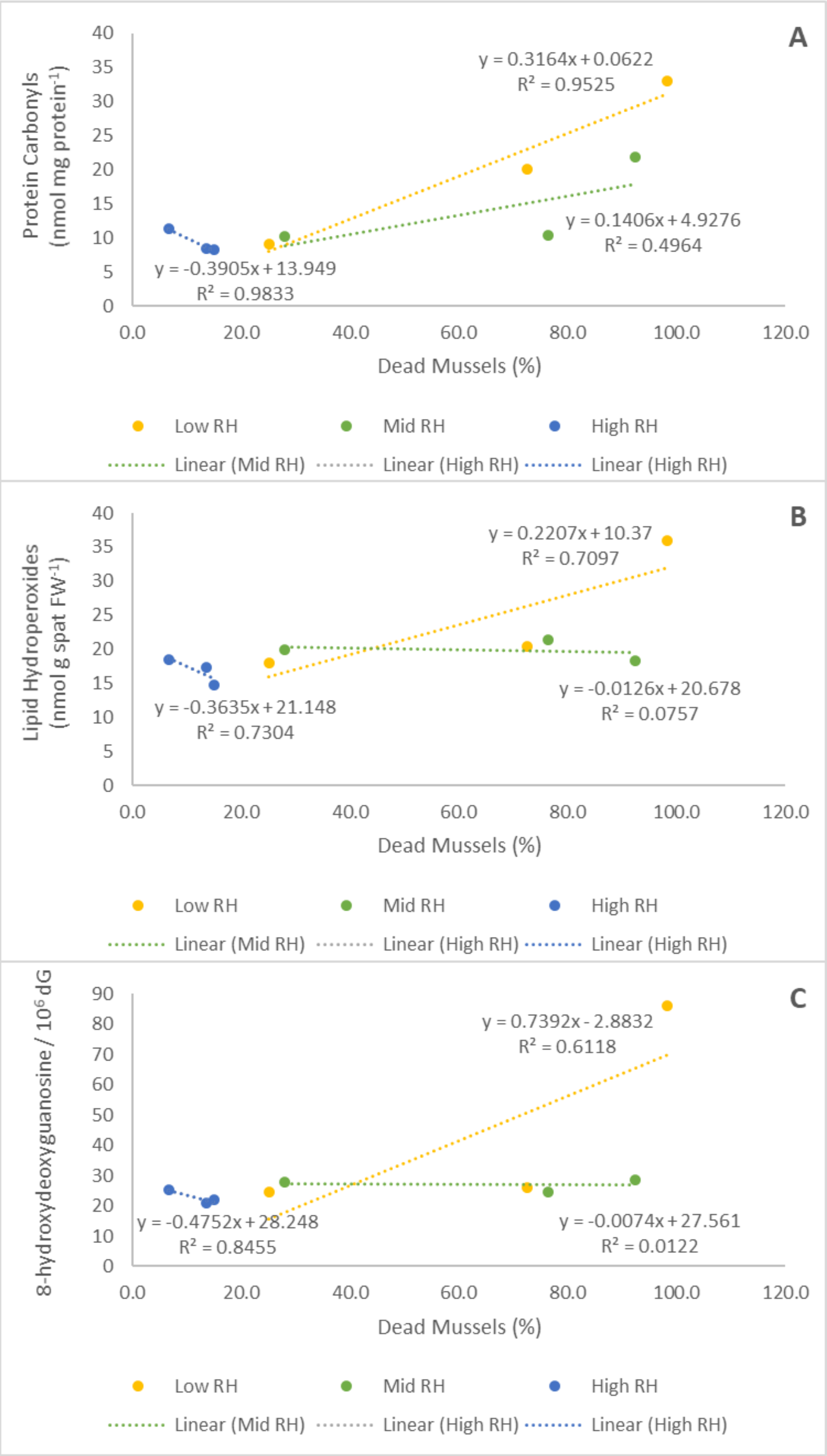

Supplement: Supplementary file 1 [file metabolites-11-00580-s001.zip › metabolites-1326783-supplementary.pdf]
